# Ulnar Collateral Ligament Reconstruction Through a Mini‐Invasive Approach and Suture Anchor Fixation

**DOI:** 10.1002/atn2.70114

**Published:** 2026-05-24

**Authors:** Valeria Vismara, Carlos Murillo Nieto, Alfonso Gonzalez‐Delgado C., Gregoire Ciais, Pierre Laumonerie

**Affiliations:** ^1^ Department of Orthopedic and Traumatology Università degli studi di Milano Milan Italy; ^2^ Department of Orthopedic Surgery Clinique Jouvenet Paris France; ^3^ Department of Orthopedic Surgery Clinique du Sport Paris France; ^4^ Department of Orthopedic Surgery Christus Muguerza Alta Especialidad Mexico City Mexico

## Abstract

Valgus stability of the elbow joint is mostly provided by the ulnar collateral ligament (UCL), which is composed of 3 bundles: anterior, posterior, and transverse. Many surgical techniques for UCL reconstruction have been described since Dr. Jobe's first introduced a technique using a free tendon graft and bone tunnel fixation on both the ulnar and humeral side. The aim of this technique is to describe a UCL reconstruction that focuses on restoring both the anterior and posterior bundle of the UCL, through a mini‐open approach, using knotless suture anchors in an onlay fashion.

## Graphical abstract

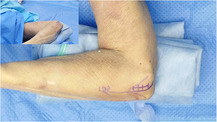

**VIDEO 1:** This technique video shows a mini‐invasive reconstruction of the ulnar collateral ligament (UCL) of the elbow using a knotless onlay construct. The procedure is performed with the patient in the supine position and the arm on a hand table, allowing fluoroscopic control throughout the case. Throughout the procedure the patient is positioned with his right arm is abducted at 70° and externally rotated. The elbow is flexed throughout the procedure from 120° to 60°, according. A soft pad is placed under the elbow. The video details key anatomic landmarks, tips to avoid ulnar nerve irritation, and strategies to minimize soft‐tissue disruption, focusing on repairing both the anterior and posterior bundle of the UCL, through a mini‐open approach, using knotless suture anchors in a onlay fashion. Video content can be viewed at https://doi.org/10.1002/atn2.70114.

Valgus stability of the elbow joint is primarily provided by the ulnar collateral ligament (UCL), which is composed of 3 bundles: anterior (aUCL), posterior (pUCL) and transverse. The aUCL is the key stabilizer between 30° and 120° of elbow flexion, whereas the pUCL contributes at higher flexion angles. UCL tears are frequent in overhead throwing athletes,^1^ as well as in acute trauma patients following elbow dislocations.^2^ Patients complain of medial‐sided elbow pain, valgus instability, and, in case of overhead throwing athletes, especially major league baseball pitchers, decreased velocity and accuracy of throws.^3^ UCL reconstruction, with or without internal brace augmentation^4^, ^5^ is often warranted for these patients and should be in the armamentarium of every elbow/sport surgeon. Meta et al. reports^6^ how the number of UCL surgeries for pitchers increased yearly from 2010 to 2023. Since the first description of UCL reconstruction by Dr. Jobe in 1974,^7^ many modifications of the technique have been proposed, mostly focusing on repairing the anterior bundle of the UCL.^8^ The aim of this technical note is to describe a mini‐open UCL reconstruction technique that preserves the epicondylar tendinous and ligamentous attachment and restores both the anterior and posterior bundles using knotless suture anchors in an onlay fashion.

## SURGICAL TECHNIQUE

### Positioning

**FIGURE 1:**
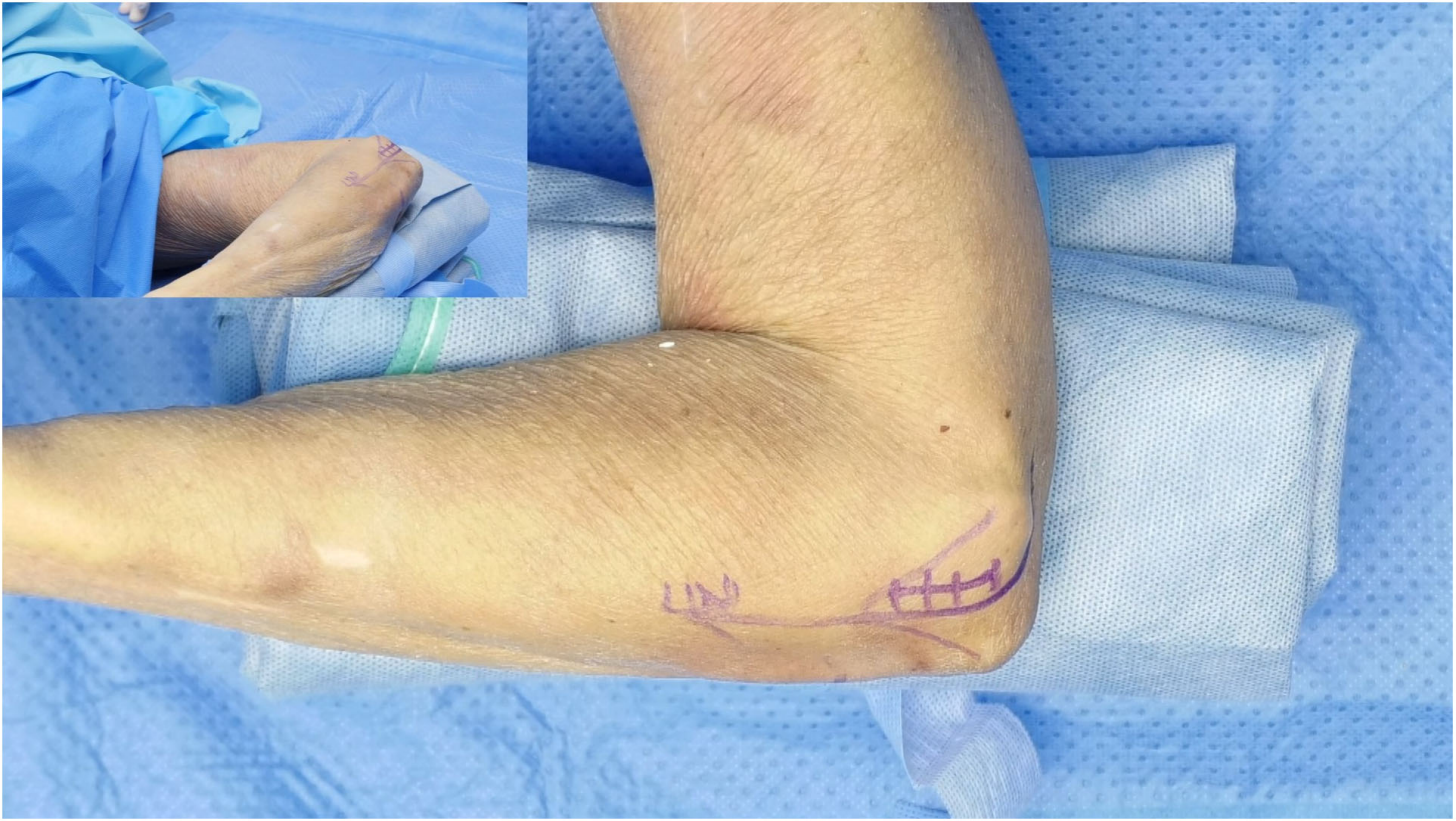
Patient positioning. The right arm is abducted at 70° and externally rotated. The elbow is flexed at 120°. A soft pad is placed under the elbow (upper left corner). The medial epicondyle and olecranon tip are marked, together with a third distal point equidistant to both, forming an isosceles triangle. The ulnar nerve course runs through the distal apex, defining a reference line. A 2‐ to 3‐cm incision line is drawn just anterior to the reference line, giving direct access to both the medial epicondylar tendons and the ulnar nerve.

The patient is placed supine with the arm on a hand table. After sterile preparation and pneumatic tourniquet inflation, the shoulder is externally rotated and abducted 70°, and the elbow flexed 110° to allow proper visualization of the medial side of the elbow joint. A soft pad beneath the elbow elevates the joint and improves posteromedial access (Figure 1, Video 1).

### Medial Exposure

The medial epicondyle and olecranon tip are marked, together with a third point 4 cm distal and equidistant to both, forming a triangle. The ulnar nerve course runs through the distal apex, defining a reference line.

**FIGURE 2:**
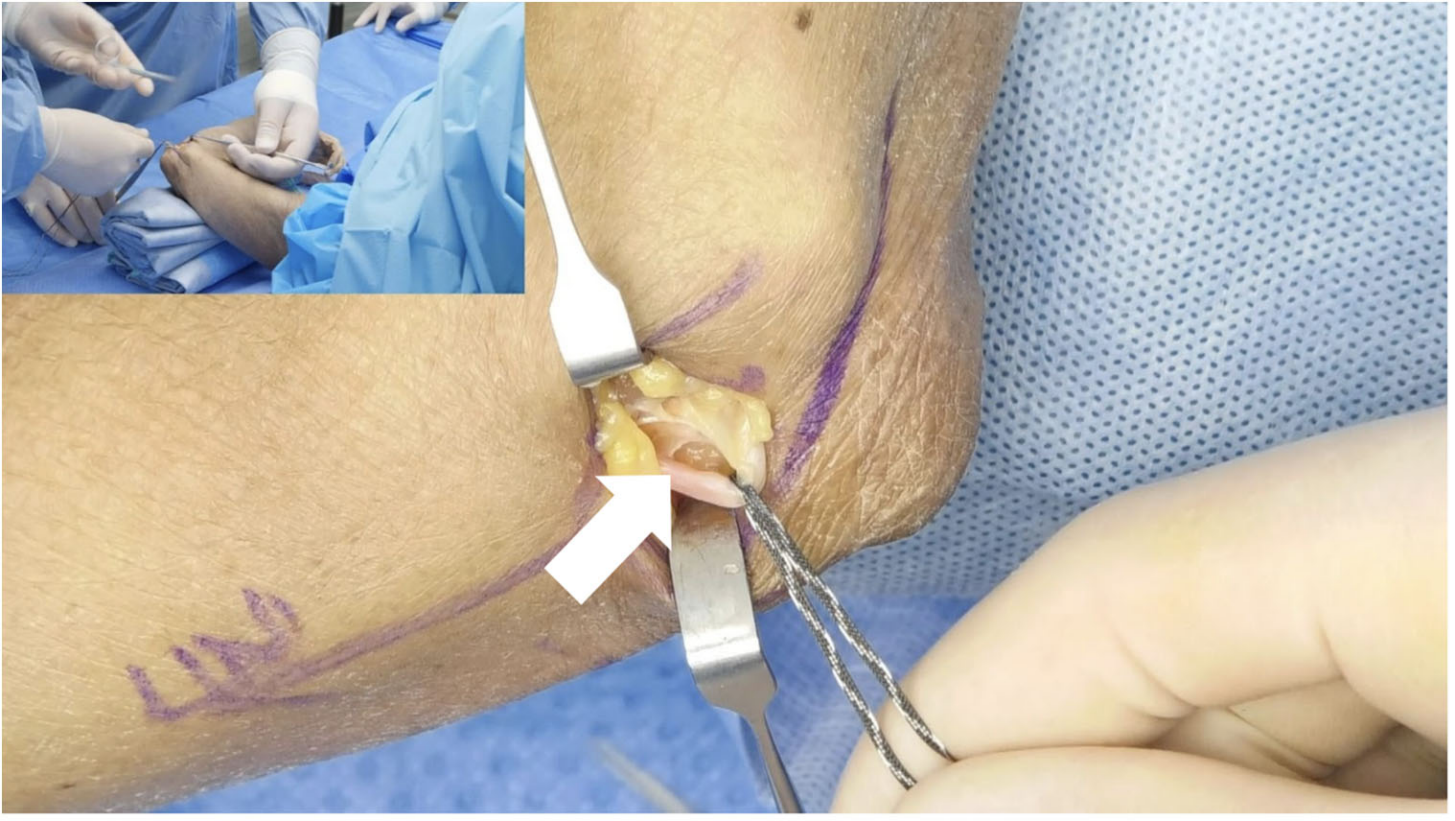
Right Elbow: right arm is abducted at 70° and externally rotated. The Elbow is flexed at 120°. A soft pad is placed under the elbow (upper left corner). Ulnar nerve (white arrow) neurolysis through flexor carpi ulnaris (FCU) split approach. A vessel loop is passed around the ulnar nerve to aid in gentle mobilization and protection of the ulnar nerve.

A 3 cm longitudinal incision is made (0.5 cm anterior to this line, marked on the patient skin) with a No. 11 Bard‐Parker scalpel blade (Aspen Surgical Products, Caledonia, MI, USA) giving direct access to the medial epicondyle while remaining safe from the ulnar nerve (Figure 1). The interval between the humeral and ulnar heads of the flexor carpi ulnaris (FCU) is identified and the fascia incised to release the ulnar nerve both distally and proximally over cubital retinaculum. A vessel loop is passed around it for protection and later management (Figure 2).

### Humeral and Ulnar Anchor Placement

**FIGURE 3:**
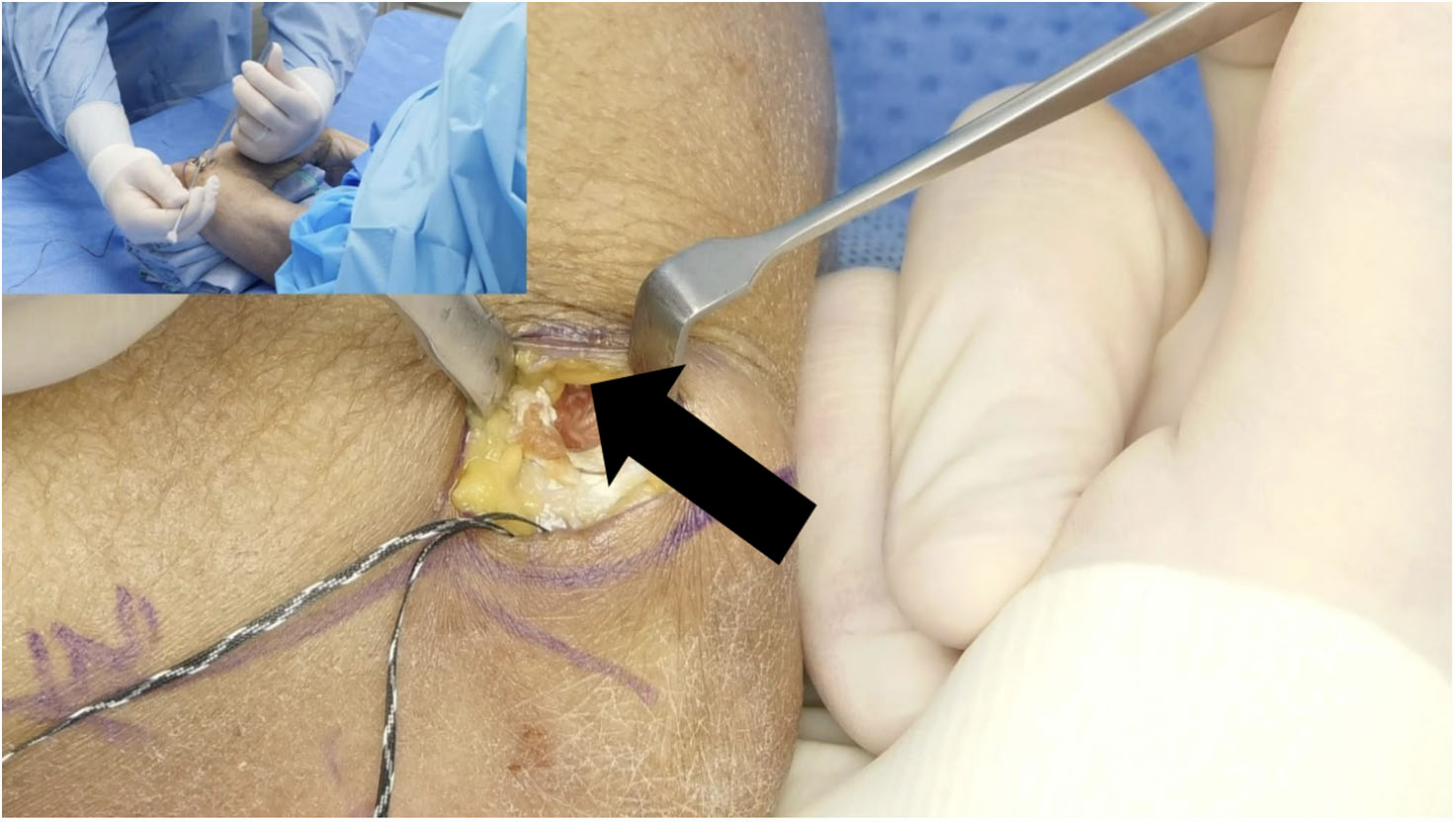
Right elbow. The right arm is abducted at 70° and externally rotated. The elbow is flexed at 120°. A soft pad is placed under the elbow (upper left corner). The fascia over the medial epicondyle is incised, along the anterior border of the medial column cortex (black arrow), just above the medial epicondylar tendons. Better exposure of the humeral cortex is obtained with the help of a Hohmann retractor on the anterior humeral cortex. The vessel loop marks the position of the ulnar nerve.

**FIGURE 4:**
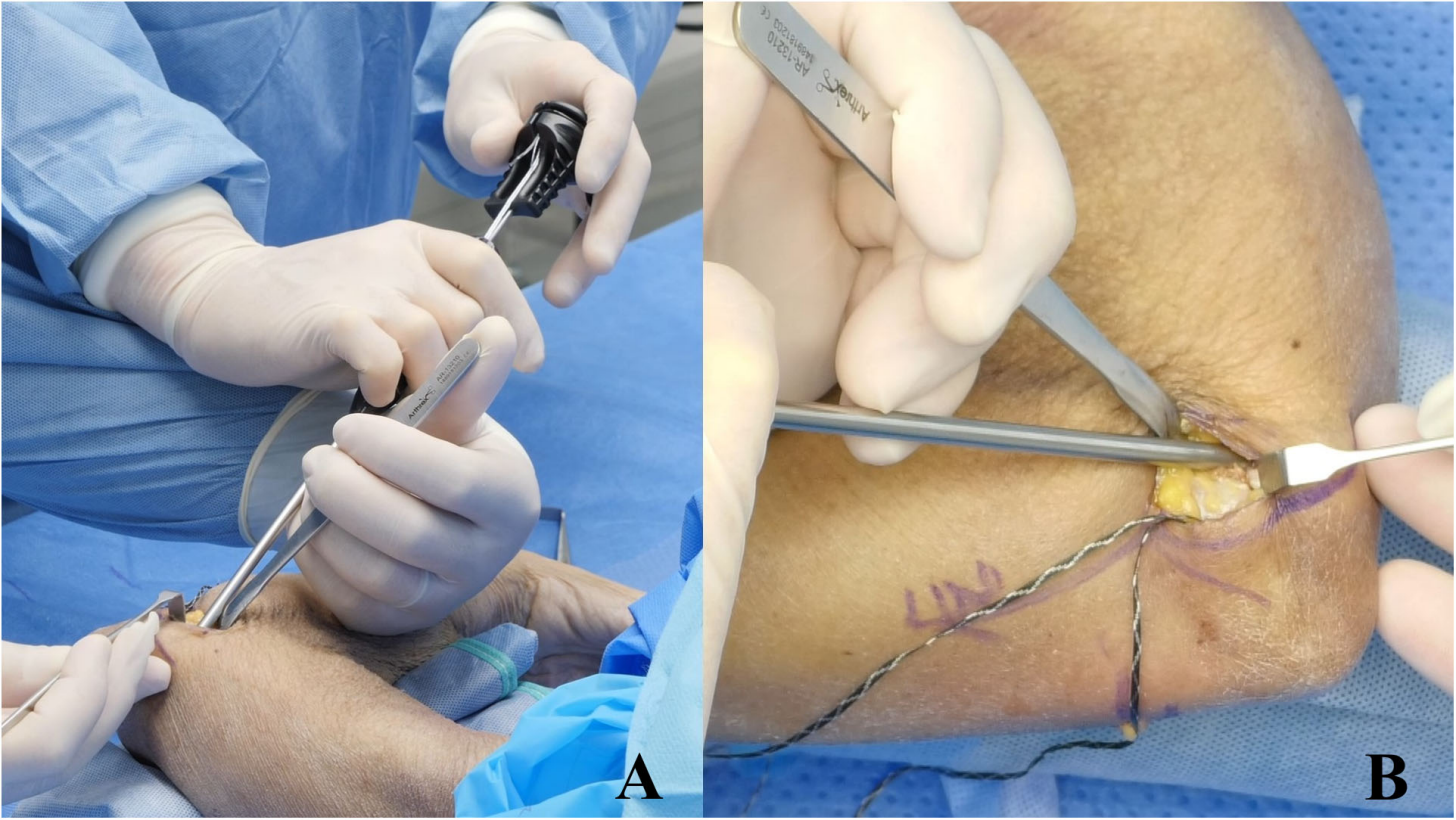
Right elbow. The right arm is abducted at 70° and externally rotated. The elbow is flexed at 120°. A soft pad is placed under the elbow. A 2.6 mm Knotless FiberTak anchor is placed perpendicular to the humeral cortex, just medial to the medial epicondyle and proximal to the anterior capsular insertion. For correct anchor placement, the anchor drill should target the posterior cortex. An oblique drilling trajectory may lead to reduced anchor stability. (A) Outside view for identifying anatomical landmark: hand, elbow, and shoulder positioning, as well as instruments positioning. (B) Close up view: the hand of the patient is superior, whereas the shoulder of the patient is medial.

Flexing the elbow 120° aligns the incision with the medial epicondylar tendons. The fatty tissue is lifted from the epicondylar tendons. Proximally, the fascia over the medial epicondyle is incised, along the anterior border of the medial column cortex, just above the medial epicondylar tendons. Better exposure of the humeral cortex is obtained with the help of a Hohmann retractor on the anterior humeral wall (Figure 3). The 2.6‐mm Knotless FiberTak anchor (Arthrex, Naples, FL) is inserted perpendicular to the cortex located at the most distal part of the medial humeral column, just medial to the medial epicondyle and proximal to the anterior capsular insertion. The anchor is advanced until it reaches the posterior cortical surface for bicortical fixation. The guide must be positioned perpendicular to the cortex to maintain a straight trajectory toward the posterior cortex, as any oblique drilling may compromise anchor stability (Figure 4).

**FIGURE 5:**
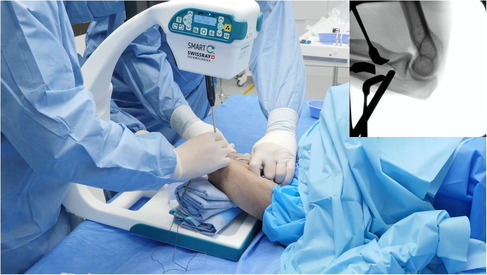
Right elbow. The right arm is abducted at 70° and externally rotated. The elbow is flexed at 120°. A soft pad is placed under the elbow. The hand of the patient is located superiorly and the shoulder of the patient is medial compared with the elbow joint. The sublime tubercle of the coronoid is exposed by retracting the humeral head of the FCU anteriorly. The ulnar nerve is posteriorly retracted. The guide for anterior ulnar anchor placement, is positioned just below the sublime tubercle, and guided by fluoroscopic control. A second 2.6‐mm Knotless FiberTak anchor is then inserted perpendicular to the cortex and placed endomedullary. Breaching the lateral cortex must be avoided to prevent radial head impingement.

Distally, the sublime tubercle of the coronoid is exposed by retracting the humeral head of the FCU anteriorly. The ulnar nerve is posteriorly retracted. The guide for anchor placement, is positioned just below the sublime tubercle, and guided by fluoroscopic control (Figure 5). A second 2.6 mm Knotless FiberTak anchor is then inserted perpendicular to the cortex and placed endomedullary. Breaching the lateral cortex must be avoided to prevent radial head impingement.

The anterior tunnel is created between the 2 anchors by dissecting the plane separating the capsuloligamentous layer from the epicondylar tendons with Stevens scissors.

### Graft Preparation and Seating

**FIGURE 6:**
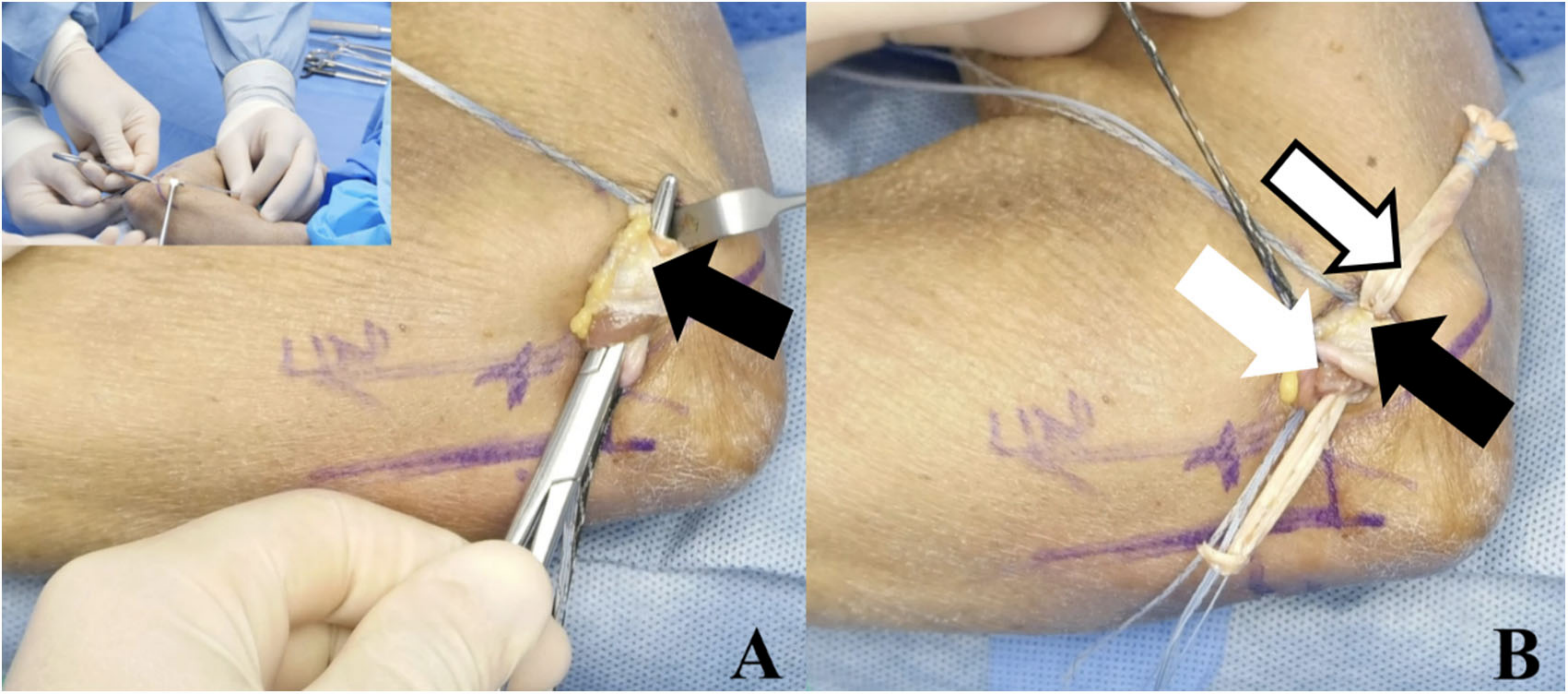
Right elbow. The right arm is abducted at 70° and externally rotated. The elbow is flexed at 120°. A soft pad is placed under the elbow (upper left corner). At close up, with the hand of the patient superior and the shoulder of the patient medial to the elbow. (A) A Kelly clamp is passed through the anterior tunnel, between the capsuloligamentous plane and the medial epicondylar ligament (black arrow). (B) The allograft (outlined white arrow) is then guided through the anterior tunnel beneath the medial epicondylar tendons (black arrow) using the Kelly clamp and subsequently passed deep to the ulnar nerve (white arrow).

A gracilis or semitendinosus allograft (≥12 cm long), either autologous or heterologous, is whip‐stitched with Krackow sutures and passed through the anterior tunnel beneath the medial epicondylar tendons and the ulnar nerve using a Kelly clamp (Figure 6).

### Reconstruction of the Anterior Bundle of the UCL (aUCL)

**FIGURE 7:**
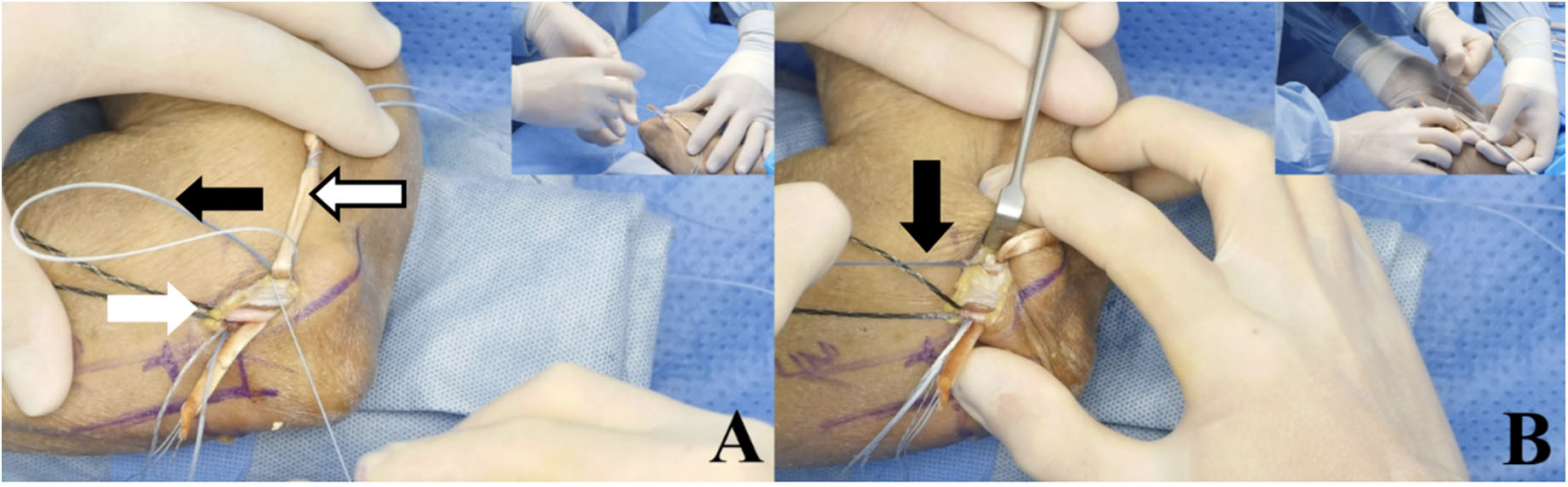
Right elbow. The right arm is abducted at 70° and externally rotated. The elbow is flexed at 120°. A soft pad is placed under the elbow (upper left corner). (A) The allograft (outlined white arrow) is then inserted into the lasso (black arrow) of the humeral FiberTak knotless, leaving approximately 5 cm proximally and 5 cm distally. The ulnar nerve (white arrow) is maintained above the graft, which passes deep to it. (B) Traction is applied along the axis of the anchor (black arrow) perpendicular to the medial column of the humerus, to ensure firm fixation of the graft against the cortex. This step provides the initial humeral fixation and defines the proximal and distal limbs of the graft for subsequent reconstruction.

Once the graft has been passed, the suture from the humeral FiberTak anchor is inserted into its shuttle loop. Pulling on the loop draws the suture into the anchor, automatically locking it and creating a knotless lasso configuration. The graft is then inserted into this lasso, leaving approximately 5 cm proximally and 5 cm distally. Traction is applied along the axis of the anchor, perpendicular to the medial column of the humerus, to ensure firm fixation of the graft against the cortex. This step provides the initial humeral fixation and defines the proximal and distal limbs of the graft for subsequent reconstruction (Figure 7).

**FIGURE 8:**
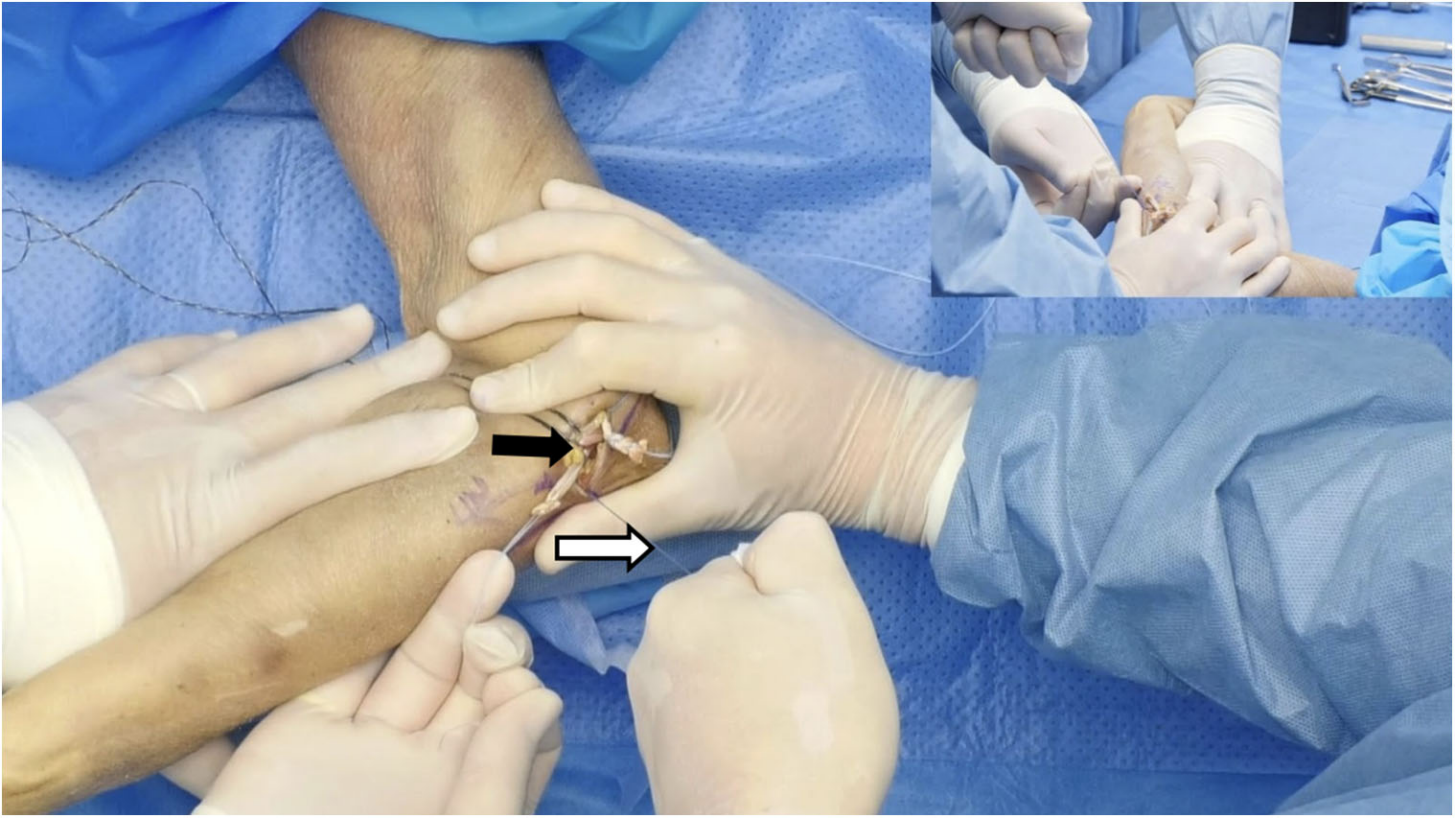
Right elbow. The hand of the patient is pointing to the lower left corner, whereas the shoulder of the patient is superiorly located. The elbow is positioned at 60° of flexion and neutral valgus. Axial traction is applied to the allograft (outlined white arrow) while pulling on the lasso loop (black arrow) to lock the fixation and seat it firmly against the ulnar cortex.

At the ulnar anchor, the same step is repeated with the elbow at 60° flexion and neutral valgus. Axial traction is applied on the allograft while locking the fixation to seat it firmly against the ulnar cortex. The elbow is cycled through full motion to confirm stable, smooth tracking without over‐tension (Figure 8).

### Reconstruction of the Posterior Bundle of the UCL (pUCL)

The free proximal graft limb, used to reconstruct the pUCL, extends from the medial epicondyle along the posteromedial ulnar cortex to the medial olecranon, providing valgus stability in deep flexion. A posterior line is drawn along the posteromedial ulnar cortex and a vertical line through the medial epicondyle; their intersection identifies the distal insertion site of the reconstructed pUCL.

**FIGURE 9:**
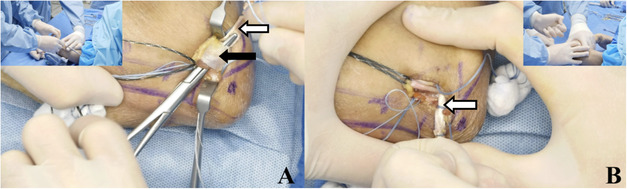
Right elbow. The right arm is abducted at 70° and externally rotated. The elbow is flexed at 120°. A soft pad is placed under the elbow (upper left corner). At close up, with the hand of the patient superior and the shoulder of the patient medial to the elbow. (A) The elbow is positioned at 110° of flexion and neutral valgus. A Kelly clamp is passed along the capsuloligamentous plane and beneath the medial epicondylar tendons (black arrow) to create a posterior tunnel and retrieve the proximal allograft limb (outlined white arrow). This latter is passed under the medial epicondylar tendons, and the ulnar nerve, and finally into the knotless lasso of a third bicortical FiberTak anchor placed bicortically into the medial ulna, vertically aligned to the epicondylar line. (B) Firm traction is applied to the graft (outlined white arrow) to achieve proper tension, and the lasso is locked by pulling perpendicular to the medial ulnar cortex, securing the graft firmly against the bone.

With the elbow flexed 110°, a third FiberTak anchor is placed bicortically into the medial ulna, vertically aligned to the epicondylar line. At this level, there is no risk of radial head impingement. The shuttling mechanism of the knotless FiberTak anchor is prepared as previously described to create the knotless lasso. A Kelly clamp is passed along the capsuloligamentous plane and beneath the epicondylar tendons to create a posterior tunnel and retrieve the proximal allograft limb. The latter is then passed first through the medial epicondylar tendons, and then under the ulnar nerve. The allograft is passed into the knotless lasso of the FiberTak anchor. Firm traction is applied to the graft to achieve proper tension, and the lasso is locked by pulling perpendicular to the medial ulnar cortex, securing the graft firmly against the bone (Figure 9).

**FIGURE 10:**
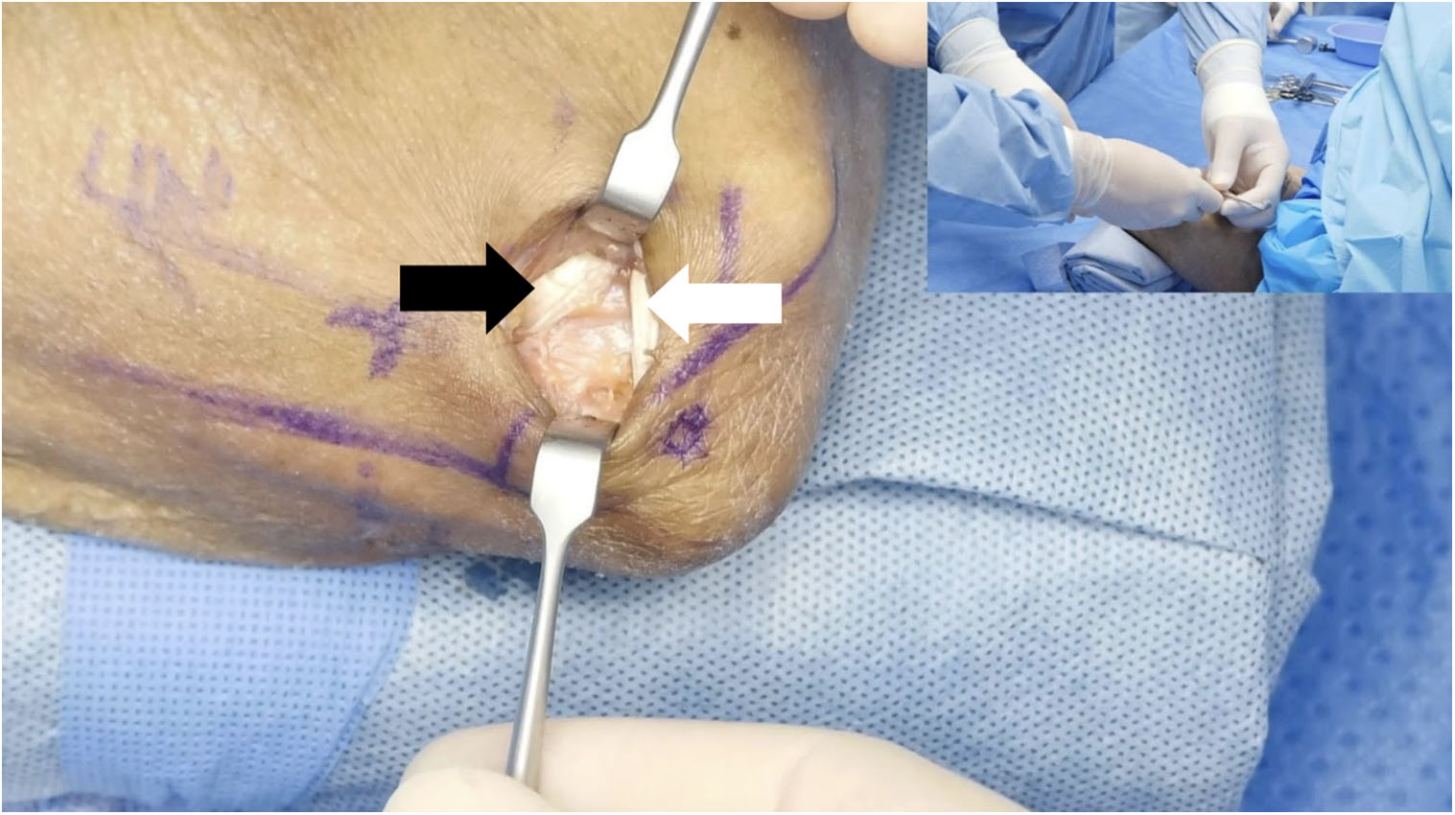
Right elbow. The right arm is abducted at 70° and externally rotated. The elbow is flexed at 120°. A soft pad is placed under the elbow (upper right corner). Final configuration of ulnar collateral ligament reconstruction, by showing both the anterior (black arrow) and posterior (white arrow) bundles of the UCL, through a cutaneous incision of 2‐3 cm.

**FIGURE 11:**
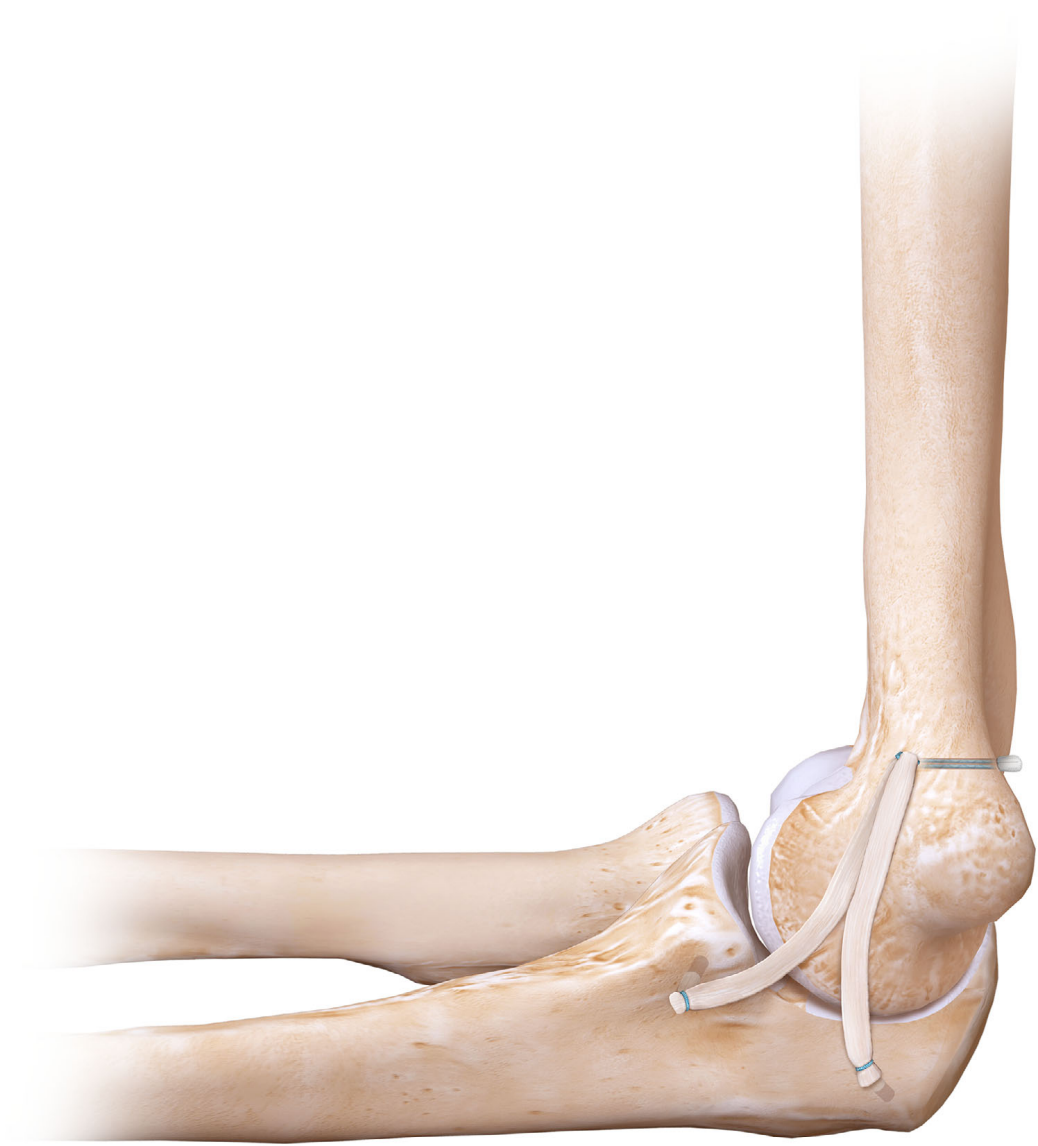
Graphical representation of the technique, in a right elbow, showing reconstruction of the anterior and posterior bundle of the ulnar collateral ligament (UCL), using a tendon graft and 3 soft tissue anchors. On the humeral side, the soft tissue anchor passes through both the anterior and posterior humeral cortex for bicortical fixation. On the ulnar side, the soft tissue anchor placed at the level of the sublime tubercle for fixation of the anterior band of the UCL is placed endomedullary, whereas the soft tissue anchor placed at the level of the olecranon for fixation of the posterior band of the UCL is placed bicortically.

All anchor suture tails and graft ends are trimmed, leaving approximately 1 to 1.5 cm beyond the locking point. Both the anterior and posterior bundles are reconstructed through a 2‐3 cm incision. Proper seating and fixation are confirmed by direct visualization and by gently cycling the elbow through its full range of motion to verify stability and anatomic tensioning. One graft limb is directed distally toward the ulnar insertion of the anterior bundle, whereas the other is oriented posteriorly to reconstruct the posterior bundle. This configuration reproduces the native fan‐shaped anatomy of the UCL, providing balanced valgus stability throughout the full arc of elbow motion (Figures 10 and 11).

Advantages and disadvantages of this surgical technique are reported in Table 1, whereas the step‐by‐step approach, together with pearls and pitfalls, are reported in Table 2.

**TABLE 1:** Advantages and Disadvantages of the Technique

**Advantages**	**Disadvantage**
Mini‐invasive approach (2 to 3 cm skin incision)	Limited exposure for the neurolysis of the ulnar nerve and for the placement of anchors
Short operative time (30‐45 minutes) with few procedural steps.	Graft failure by anchor pull out
Reconstruction of both anterior and posterior band of the UCL	Graft failure due to a “suture slippage” from the lasso configuration of knotless anchors and axial traction on the graft
Preservation of the medial epicondylar tendons	Risk of radial head impingement if the distal anchor for anterior UCL is placed beyond the lateral ulnar cortex
Simplified graft tensioning and adjustment with onlay fixation	Lower bone‐to‐graft contact area compared with inlay constructs
No ulnar nerve transposition	Non‐anatomic anchor placements relative to the native UCL footprints
Low risk of cortical weakening compared with inlay construct	
Enables early rehabilitation including immediate active motion	

UCL, ulnar collateral ligament.

**TABLE 2:** Step‐by‐Step Technique and Pearls and Pitfalls of the Technique

Step‐by‐Step Technique	Pearls and Pitfalls
**Patient Positioning** • The patient is positioned supine with hand table extension • Pneumatic tourniquet is applied on the upper arm • The upper extremity is prepared in the sterile fashion • Pneumatic tourniquet is insufflated at 250 mm/hg • The shoulder is rotated externally and abducted at 70° • The elbow is flexed 110°	• Pearl: A soft pad is placed under the elbow to open more the posterior‐medial side and have a better visualization of the soft tissue during surgery
**Medial Exposure** • Medial epicondyle and tip of the olecranon are identified. A third point that is located 4 cm equally distant to the tip of the olecranon and medial epicondyle. Thus, an isosceles triangular shape is formed • Ulnar nerve path is mark and passes through the distal apex of the marked triangle • A 3 cm longitudinal incision is made, 0.5 cm anterior of the passage of the marked ulnar nerve • Blunt dissection trough subcutaneous fat tissue • The plane between humeral and ulnar head of the flexor carpi ulnaris (FCU) is identified • The fascia between the 2 heads of the FCU is opened for ulnar nerve neurolysis for complete mobilization	• Pearl: The ulnar nerve should be always identified and protected. • Pearl: A vessel loop can be used for gentle traction and mobilization of the ulnar nerve as well as for nerve identification throughout the procedure.
**Humeral Attachments for Graft** • The elbow is flexed to 120° to align the incision with the medial epicondylar tendons. • Proximally, the fascia along the anterior border of the medial column cortex, just above the medial epicondylar tendons is incised. • A Hohmann retractor is placed to better expose the anteroinferior surface of the medial column • The 2.6‐mm Knotless FiberTak anchor is inserted perpendicular to the cortex located at the most distal part of the medial humeral column, just medial to the medial epicondyle and proximal to the anterior capsular insertion. • The anchor is advanced until it reaches the posterior cortical surface for bicortical fixation. • The suture from the humeral FiberTak anchor is inserted into a shuttle loop • Pulling on the loop draws the suture into the anchor, automatically locking it and creating a knotless lasso configuration.	• Pearl: For optimal exposure, Hohmann retractor is placed to better identify the medial column of the humerus and its medial border. • Pearl: It is essential to keep the guide perpendicular to the cortex and to insert the anchor aiming for the posterior cortex. • Pitfall: An oblique drilling trajectory may lead to reduced anchor stability. • Pearl: Knotless FiberTak fixation is bicortical
**Ulna Attachment for Anterior Graft** • The ulnar nerve is retracted posterior meanwhile FCU anteriorly • The guide for anchor placement, is positioned just below the sublime tubercle, and guided by fluoroscopic control • A 2.6‐mm Knotless FiberTak anchor is then inserted perpendicular to the ulna cortex and placed endomedullary. • The suture from the ulna knotless FiberTak anchor is inserted into a shuttle loop • Pulling on the loop draws the suture into the anchor and creates a knotless lasso configuration • A space between the capsuloligamentous plane (deep layer) and the medial epicondylar tendons (superficial layer) is dissected to create an anterior tunnel connecting the humeral and ulnar anchors.	• Pearl: Fluoroscopy is used to obtain a true lateral view of the elbow to confirm the position of the guide in relation to the sublime tubercle • Pearl: The Knotless FiberTak anchor should be inserted perpendicular to the cortex to ensure solid fixation • Pearl: Knotless FiberTak fixation is unicortical with the anchor placed endomedullary without breaching the lateral ulnar cortex to prevent radial head impingement.
**Anterior UCL Reconstruction** • The allograft should be prepared at both ends with suture using a Krackow technique • The allograft is then inserted into this lasso, leaving approximately 5 cm proximally and 5 cm distally • Traction is applied along the axis of the anchor, perpendicular to the medial column of the humerus, to ensure firm fixation of the graft against the cortex. • The graft is pass through the anterior tunnel previously prepared from proximal to distal • The graft is passed under the ulnar nerve and into the lasso loop from the ulna • The elbow should be flexed at 60° and neutral position • Axial traction is applied on the allograft while locking the fixation to seat it firmly against the ulnar cortex	• Pearl: A gracilis tendon or semitendinosus tendon auto/allograft can be used for UCL reconstruction • Pitfall: Care is taken during this step to avoid traction or compression of the nerve, ensuring that the graft remains entirely underneath the ulnar nerve along its course. • Pearl: Before tensioning the distal end of the graft at the sublime tubercle, the elbow is placed at 60° of flexion with neutral valgus.
**Posterior UCL Reconstruction** • A 2.6‐mm Knotless FiberTak anchor is inserted bicortically into the ulna, vertically aligned to the epicondylar line • The shuttling mechanism of the device is prepared as before and creates a knotless lasso configuration • A space between the capsuloligamentous plane (deep layer) and the medial epicondylar tendons (superficial layer) is dissected to create the posterior tunnel connecting the humeral and the posterior ulnar anchors. • A Kelly clamp is placed through the posterior tunnel to retrieve the proximal graft end, which is passed under the medial epicondylar tendons, then under the ulnar nerve. • The allograft is placed through in the knotless lasso loop of the posterior ulnar anchor • The elbow is then flexed to 110°. • A strong distal traction is applied along the graft axis, and the lasso is locked by pulling perpendicular to the ulnar cortex • The sutures and graft ends are trimmed • Both of the aUCL and pUCL bundle are reconstructed trough 2 to 3 cm incision in the elbow	• Pearl: The elbow is then flexed to 110° before locking the lasso of the posterior ulnar anchor • Pearl: Knotless FiberTak anchor is inserted bicortically into the medial cortex of the ulna because there is no risk of radial head impingement • Pearl: The ulnar nerve is carefully maintained above the graft throughout the procedure • Pearl: Full elbow range of motion should be tested before closure, to be sure of lack of over or under tensioning of the graft and lack of irritation or compression around the ulnar nerve.

aUCL, anterior UCL; FCU, flexor carpi ulnaris; pUCL, posterior UCL; UCL, ulnar collateral ligament.

### Postoperative Care

Postoperative care follows the “Fix it, Move it” principle: no immobilization, immediate cryocompression therapy and active motion, with progressive stretching and strengthening of the medial epicondylar muscles, while avoiding passive mobilization. Return to light sports is allowed at 1.5 months and return to high‐demand activities is permitted at 2‐3 months.

## DISCUSSION

Since Dr. Jobe's original description, numerous surgical techniques for UCL reconstruction have been proposed,^2^ most of which focus primarily on the anterior band as the main valgus stabilizer.^9^ The main complications of UCL reconstruction include joint stiffness, arthrofibrosis requiring manipulation under anesthesia, graft failure, ulnar neuropathy, and loss of strength or range of motion.

The presented knotless, minimally invasive onlay reconstruction aims to restore the native fan‐shaped configuration of the UCL while preserving the integrity of the medial epicondyle and its musculotendinous attachments. Recent biomechanical studies have highlighted the critical role of the medial epicondyle as both the proximal attachment of the UCL and the origin of the flexor‐pronator mass, forming the central link between static and dynamic stabilizer.^10^, ^11^ The epicondylar region provides essential valgus and rotational control between 30° and 90° of flexion. Even partial detachment or devascularization of the medial epicondyle significantly compromises this equilibrium, leading to increased posteromedial joint pressures, valgus laxity, and a higher risk of subluxation, osteophyte formation, and progressive arthrosis.^10^, ^11^

By avoiding detachment of the flexor‐pronator mass and eliminating the need for ulnar nerve transposition, this approach could reduce the risk of stiffness, ulnar neuropathy, and complications related to devascularization or excessive dissection. The ulnar anchors are placed between the 2 heads of the FCU, the humeral anchor is positioned just beyond the medial epicondylar tendons, and the graft tunnel passes between the capsule and the flexor‐pronator mass, maintaining the native anatomic relationships.

In contrast to techniques relying on bone tunnels,^9^, ^12^ this onlay construct minimizes bone weakening and the risk of fracture or graft failure. Extending reconstruction to not only the anterior but also the posterior bundle allows to restore stability in mid‐flexion (30°‐60°) and prevents posteromedial subluxation in complex valgus‐instability patterns.^13^ This combined, knotless reconstruction provides a biomechanically robust, anatomic, and biologically favorable solution. It allows broad graft‐bone contact for enhanced healing, reproduces the natural valgus constraint throughout the motion arc, and facilitates an early, functional recovery. Future biomechanical and clinical studies should assess comparative stability, load‐to‐failure, and return‐to‐sport outcomes, while further elucidating potential failure mechanisms such as anchor pull‐out or graft attenuation, through the suture slippage in the knotless suture anchor.
